# A differentially-methylated-region signature predicts the recurrence risk for patients with early stage lung adenocarcinoma

**DOI:** 10.18632/aging.206139

**Published:** 2024-11-18

**Authors:** Heng Li, Fuchao Luo, Xiaoran Sun, Chunhua Liao, Guoqiang Wang, Yusheng Han, Leo Li, Chunwei Xu, Wenxian Wang, Shangli Cai, Gao Li, Di Wu

**Affiliations:** 1 Yunnan Cancer Hospital and The Third Affiliated Hospital of Kunming Medical University and Yunnan Cancer Center, Kunming, P.R. China; 2 Chongqing University Fuling Hospital, Chongqing, P.R. China; 3 Burning Rock Biotech, Guangzhou, P.R. China; 4 Institute of Basic Medicine and Cancer, Chinese Academy of Sciences, Hangzhou, P.R. China; 5 The Cancer Hospital of the University of Chinese Academy of Sciences, Hangzhou, P.R. China; 6 Hainan General Hospital, Hainan Affiliated Hospital of Hainan Medical University, Haikou, P.R. China; 7 The First Affiliated Hospital of Southern University of Science and Technology (Shenzhen People’s Hospital), Shenzhen, P.R. China

**Keywords:** lung adenocarcinoma, DNA methylation, prognosis, recurrence

## Abstract

Predicting prognosis in lung cancer patients is important in establishing future treatment and monitoring plans. Lung adenocarcinoma (LUAD) is the most common and aggressive type of lung cancer with dismal prognosis and prognostic stratification would help to guide treatment. Aberrant DNA methylation in tumors occurs earlier than clinical variations, and keeps accumulating as cancer progresses. Preliminary studies have given us some clues that DNA methylation might serve as a promising biomarker for prognosis prediction. Herein, we aimed to study the potential utility of DNA methylation pattern in predicting the recurrence risk of early stage resectable LUAD and to develop a risk-modeling signature based on differentially methylated regions (DMRs). This study consisted of three cohorts of 244 patients with stage I–IIIA LUAD, including marker discovery cohort (*n* = 39), prognostic model training cohort (*n* = 117) and validation cohort (*n* = 80). 468 DMRs between LUAD tumor and adjacent tissues were screened out in the marker discovery cohort (adjusted *P* < 0.05), and a prognostic signature was developed based on 15 DMRs significantly related to disease-free survival in early stage LUAD patients. The DMR signature showed commendable performance in predicting the recurrence risk of LUAD patients both in model training cohort (*P* < 0.001; HR = 4.32, 95% CI = 2.39–7.80) and model validation cohort (*P* = 0.009; HR = 9.08, 95% CI = 1.20–68.80), which might be of great utility both for understanding the molecular basis of LUAD relapse, providing risk stratification of patients, and establishing future monitoring plans.

## INTRODUCTION

Lung cancer stands as the primary cause of cancer-related fatalities, exhibiting a dismal 5-year survival rate of merely 22%, as reported [1]. Lung adenocarcinoma (LUAD), a highly aggressive subtype of non-small cell lung cancer (NSCLC), prevails as the most prevalent type, accounting for approximately 40% of all lung cancer cases [2]. LUAD originates from the small airway epithelia and type II alveolar cells, which function in secreting mucus and other vital substances [2, 3]. In the case of early-stage NSCLC, surgical resection is widely deemed as the most effective and curative treatment modality. Nevertheless, despite complete resection, approximately half of patients with stage I–IIIA NSCLC experience recurrence and succumb within five years [4, 5]. Notably, a prior study revealed that postoperative relapse is the leading cause of mortality post-surgery [6], thereby emphasizing the clinical significance of assessing the individual patient’s risk of postoperative recurrence.

The occurrence of molecular alterations in tumors precedes clinical manifestations, enabling effective and novel molecular biomarkers to precisely forecast the prognosis of patients and cancer recurrence. Furthermore, these biomarkers hold the potential in tailoring individualized treatment plans. Notably, all human cancers exhibit epigenetic aberrations, with cancer onset and progression being a culmination of genetic variations, epigenetic modifications, and environmental influences [7]. Among epigenetic alterations, DNA methylation safeguards against recombination events between repetitive sequences, thereby suppressing gene activity and maintaining genomic stability [8]. Cancer cells exhibit an abnormal methylation pattern, manifesting either as specific hypermethylation or widespread hypomethylation on the promoter of tumor suppressor genes [8, 9]. Numerous aberrant DNA methylation signatures, particularly those related to gene-specific promoter methylation, have also been discerned in lung cancer [10]. During the progression from a normal lung to atypical adenomatous hyperplasia and ultimately adenocarcinoma, a range of genes crucial for various cellular functions undergo epigenetic disruption. These include *CDKN2A*, *MGMT* (DNA repair gene), *DAPK* (alteration of apoptosis), *RASSF1A* (Ras signaling), *RARb* (retinoic acid signaling), and *hTERT* (immortalization). These hallmark genes, along with others, undergo epigenetic modifications at distinct phases of LUAD carcinogenesis [11–15]. Notably, *CDKN2A* emerged as the pioneering tumor suppressor gene discovered to be deactivated in lung cancer, predominantly via aberrant hypermethylation mechanisms [16]. Besides function in carcinogenesis, the inactivation of metastasis suppressor genes, exemplified by *CDH11*, assumes a pivotal role in the process of pulmonary metastasis [17].

Predicting prognosis in lung cancer patients is paramount for devising tailored treatment and surveillance strategies. While TNM staging has traditionally served as a prognostic indicator, recent advancements have incorporated molecular markers like EGFR, ALK, and PD-L1. Additionally, aberrant DNA methylation in lung cancer has emerged as a promising biomarker for prognostic assessment. Studies evaluating the prognostic value of methylation in lung cancer predominantly focused on patients with early-stage disease who underwent surgery, utilizing methylation-specific polymerase chain reaction to target methylation changes in known gene promoters. The methylation of promoter regions within a four-gene panel, comprising *APC, RASSF1A, p16*, and *CDH13*, in surgically treated patients with stage I NSCLC was found to be associated with early tumor recurrence [18]. This groundbreaking study is significant as it presents the first evidence that DNA methylation markers can independently predict lung cancer prognosis, setting itself apart from traditional prognostic factors. It has also been reported that methylation in *p16* and *CDH13* serves as an effective prognostic biomarker in LUAD [19]. The hypermethylation of tumor suppressor gene promoters is predominantly linked to adverse outcomes. The concurrent methylation of more than four tumor suppressor genes was associated with inferior 2-year progression-free survival (PFS) in matched samples of NSCLC tumor tissue and adjacent normal tissue [20]. Specifically, the promoter methylation of *BRMS1* was linked to decreased disease-free survival (DFS) among 325 NSCLC patients undergoing surgical intervention [21]. Furthermore, elevated methylation levels of *SHOX2* and *PITX2* emerged as significant predictors of PFS in NSCLC patients who underwent surgical treatment [22]. Furthermore, through DNA methylation profiling utilizing microarrays encompassing 27,578 cytosine-phosphate-guanine (CpG) sites, a specific gene set comprising 10 CpGs in 10 genes was associated with better prognosis in 48 patients with stage I NSCLC [23]. Moreover, 33 DNA methylation sites were identified as novel biomarkers of prognosis and recurrence and therapeutic targets for LUAD [24].

Preliminary studies pinpointed promising biomarkers for predicting the prognosis of early lung cancer based on aberrant methylation changes across diverse samples, anticipating that novel insightful methodologies will furnish even more valuable prognostic and predictive marker data. Since differentially methylated regions (DMRs) comprising multiple CpG sites have been shown to hold greater significance in cancer detection than individual CpG sites as reported in the literature [25], we have designated CpG sites that exhibit close genomic proximity and high correlation in their methylation levels as specialized methylation blocks. Herein, we aimed to explore the potential utility of DNA methylation pattern in predicting the recurrence risk of early stage resectable LUAD and to develop a risk-modeling signature based on DMRs.

## MATERIALS AND METHODS

### Patient and study design

The study consisted of three cohorts, including marker discovery cohort (*n* = 39), prognostic model training cohort (*n* = 117) and prognostic model validation cohort (*n* = 80). All eligible patients with stage I–IIIA LUAD were enrolled from the First Affiliated Hospital of Southern University of Science and Technology. Tumor tissues and adjacent lung tissues were collected during surgical resection. The diagnosis of LUAD was founded upon the assessment of a pathological specimen subsequent to surgical resection. Researchers assigned pathological stages to all patients, adhering to the 8th edition of the American Joint Committee on Cancer (AJCC) classifications. Patients identified with stage IIIB/IV lung cancer, other types of cancer, anaemia, or autoimmune diseases were excluded from the study. LUAD samples with a tumor fraction less than 40% were excluded from the study. This study was conducted in accordance with the Declaration of Helsinki and approved by the Institutional Review Board of the First Affiliated Hospital of Southern University of Science and Technology (AUP-220516-CHR-0390-01). Written informed consents were obtained from all patients. The clinicopathologic characteristics of the three cohorts included in this study are summarized in Table 1. The baseline characteristics including age, sex, smoking history and TNM stage were collected for all the three cohorts and the survival was collected for the model training and validation cohorts only.

**Table 1:** Baseline characteristics of the cohorts included in the study.

**Characteristics**	**Marker discovery cohort** **(*n* = 39)**	**Model training cohort** **(*n* = 117)**	**Model validation cohort** **(*n* = 80)**
**Sex**			
Male	24 (61.5%)	61 (52.1%)	39 (48.8%)
Female	15 (38.5%)	56 (47.9%)	41 (51.2%)
**Age**			
<65 yrs	26 (66.7%)	83 (70.9%)	42 (52.5%)
≥65 yrs	13 (33.3%)	34 (29.1%)	38 (47.5%)
**Smoking history**			
Yes	21 (53.8%)	60 (51.3%)	25 (31.3%)
No	17 (43.6%)	57 (48.7%)	55 (68.7%)
NA	1 (2.6%)		
**AJCC stage**			
I	39 (100.0%)	46 (39.3%)	60 (75.0%)
II		28 (23.9%)	7 (8.7%)
IIIA		43 (36.8%)	13 (16.3%)

The marker discovery cohort was used to identify LUAD-specific DMRs, the prognostic training cohort was used to develop a risk-modeling signature based on the above DMRs and the prognostic validation cohort was used to validate the developed signature.

### Target methylation sequencing and data preprocessing

The procedure for DNA extraction was as previously described [26]. In summary, for tissue samples, the DNA extraction process was carried out utilizing the QIAamp DNA formalin-fixed and paraffin-embedded tissue kit, adhering rigorously to the manufacturer’s prescribed instructions. Subsequently, DNA concentration was quantified using the Qubit double-stranded DNA assay (Life Technologies, Carlsbad, CA, USA).

As for methylation sequencing, a capture-based approach was employed to identify CpG sites. The bisulfite sequencing library was constructed utilizing the brELSATM methodology (Burning Rock Biotech, Guangzhou, China) [27]. Subsequently, the targeted libraries underwent quantitative analysis through real-time PCR and were sequenced on NovaSeq 6000 achieving an average target depth of 500×. With the raw sequencing data, a suite of bioinformatics tools, encompassing Trimmomatic, BWA-meth, and samblaster were deployed to facilitate read alignment and variant calling as part of the downstream analytical pipeline. Notably, as reported, DMRs comprising multiple CpG sites played more pivotal roles in cancer detection compared to individual CpG sites [28]. Methylation blocks were designated as CpG sites exhibiting close genomic proximity and a high degree of correlation in their methylation levels. Utilizing the 80,672 CpG sites, a total of 8,312 such blocks were identified. The score for each block was calculated, incorporating both the depth of coverage and the distance between adjacent CpG sites as follows [29]:


$$Methylation\;Block\;Score=\frac{1}{n}\times \sum_{i=1}^{n}\left(\frac{\sum_{i=1}^{m}l_{ij}^{2}}{L_{i}^{2}}\right)$$

For a specified methylation block, *n* represents the aggregate number of reads encompassing several CpG sites, and *L_i_* designates the number of CpG sites covered on *i*th read. The length of consecutive methylated CpG sites exceeding 1 is denoted as *l_ij_*, and *m* signifies the total counts on *i*th read. To normalize the depth variation, the number of reads within each block is employed, thereby constraining the metric within the range of 0 to 1.

### Marker selection for LUAD

DMRs between LUAD samples and matched adjacent tissue samples were identified in the marker discovery cohort (LUAD: *n* = 39; matched normal tissue: *n* = 39) with the cutoff values determined as the absolute value of mean difference of methylation levels >0.1 and adjusted *P*-value < 0.05.

### Construction and validation of a DMR signature related to recurrence

To screen DMRs related to the DFS of LUAD patients in the model training cohort (*n* = 117), a univariable Cox regression analysis was employed. To minimize the risk of overfitting, we used the Least Absolute Shrinkage and Selection Operator (LASSO) penalized Cox regression analysis to construct a prognostic risk model. The LASSO algorithm selects and contracts the variables through R package “glmnet” so that some of the regression coefficients are strictly equal to zero, resulting in an interpretable model. The risk score of each patient was calculated according to the methylation values of selected DMRs and the corresponding regression coefficients:


$$Risk\;score=\sum_{i-1}^{n}MBS_{i}\times Coef_{i}$$

For each patient, *n* represents the number of DMRs with prognostic value, *MBS_i_* is the methylation block score of DMR *i*, and *Coef_i_* represents the regression coefficient of DMR *i*.

**Figure 1:**
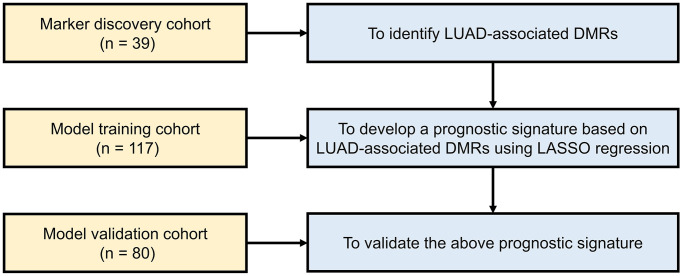
**Work flow of the study.** Abbreviations: LUAD: lung adenocarcinoma; DMR: differentially methylated region; LASSO: least absolute shrinkage and selection operator.

Patients were categorized into high-risk and low-risk groups utilizing the risk score threshold determined from the training set. Survival analysis was performed by R packages “survival” and “survminer” to analyze DFS between the high-risk and low-risk patients. To assess the predictive accuracy of the prognostic DMR signature, a time-dependent receiver operating characteristic (ROC) curve analysis was conducted with “timeROC” R package. Both univariable and multivariable Cox regression analyses were performed, aiming to uncover the independent prognostic significance of the risk score. In addition, the calculation of risk score, grouping of samples, survival analysis, ROC analysis, and univariable and multivariable Cox regression analyses were also performed in the validation cohort (*n* = 80) for independent validation. The study design is shown in Figure 1 by a flow chart.

### Comparison of risk score between clinical subgroups

Samples in both model training and validation cohorts were divided into subgroups according to several clinical characteristics including sex, age, smoking history, and TNM stage. The comparison of risk scores, derived from the DMR signature, was conducted between different subgroups.

### Functional enrichment analysis

Gene enrichment analysis targeting the biological process and biological pathways in the genes corresponding to the DMRs was via the “clusterProfiler” R package combined with Gene Ontology (GO) and Kyoto Encyclopedia of Genes and Genomes (KEGG) database.

### Statistical analysis

R software (version 4.2.0; https://www.R-project.org) was used for all statistics. Wilcoxon test was used to screen DMRs. Kaplan-Meier (KM) curves were drawn, and the difference of DFS between risk groups was tested by Log-rank test. The area under ROC curve (AUC) was used to judge the accuracy of predicting. The comparison of risk score between different clinical subgroups was via Wilcoxon test. For all analyses in the study,* P* < 0.05 was considered statistically significant.

### Data availability statement

The relevant data supporting the findings of this study are available within the paper. Due to ethical and privacy concerns, we are unable to publish the patient-level data in our study, of which readers may contact the corresponding authors for access for non-commercial purposes.

## RESULTS

### Identification of DMRs in early stage LUAD patients

**Figure 2:**
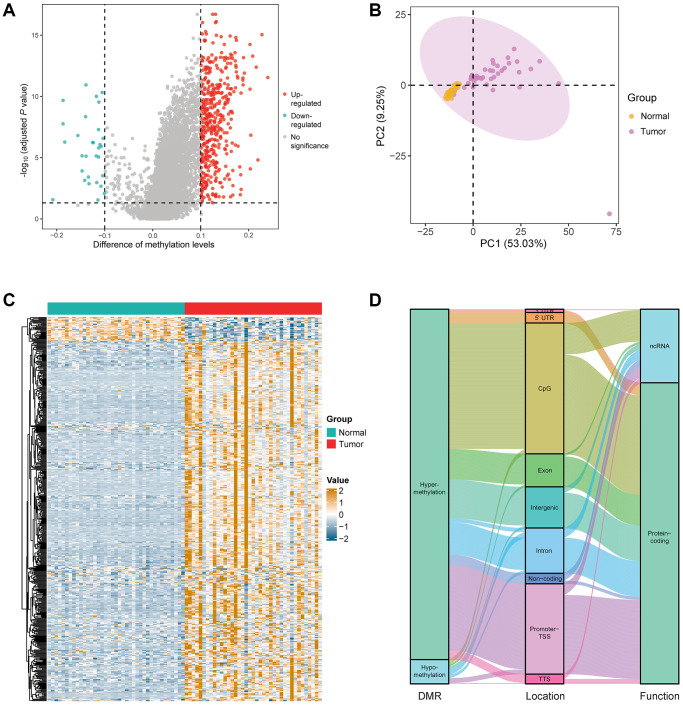
**Identification of DMRs in the patients with early stage LUAD.** (**A**) Difference analysis yielded a total of 468 DMRs, including 438 hypermethylated regions and 30 hypomethylated regions in the marker discovery cohort (LUAD: *n* = 39; normal: *n* = 39). (**B**) Principal component analysis between LUAD and normal lung samples based on the methylation value of 468 DMRs. (**C**) Heatmap of the 468 DMRs in the marker discovery cohort. (**D**) Annotation information of the 468 DMRs, including location and the function of corresponding genes.

In order to better reflect the LUAD-associated methylation alteration, we first tried to identify DMRs in LUAD by comparing 39 stage I LUAD samples and 39 matched adjacent lung tissue samples in the marker discovery cohort. Investigation of DMRs between the two groups yielded a total of 468 DMRs, which included 438 hypermethylated regions and 30 hypomethylated regions (Figure 2A and Supplementary Table 1). Principal component analysis showed an obvious difference between LUAD tumor and adjacent tissues based on the methylation value of 468 DMRs (Figure 2B). Heat map of the 468 DMRs shows the hypo- and hypermethylated regions in LUAD compared with adjacent lung tissues (Figure 2C). Most of the DMRs located in the CpG islands and transcription start site of promoter (59.1%), and contributed to protein-coding (80.4%) (Figure 2D). In addition, the genes corresponding to the DMRs were mostly enriched in the biological processes including cell fate specification, cell fate commitment, pattern specification process, embryonic organ morphogenesis, and embryonic skeletal system morphogenesis (Supplementary Figure 1A), and the signaling pathways including transcriptional misregulation in cancer, maturity-onset diabetes of the young, and cAMP signaling pathway (Supplementary Figure 1B).

### Construction of a 15-DMR prognostic signature in early stage LUAD patients

**Figure 3:**
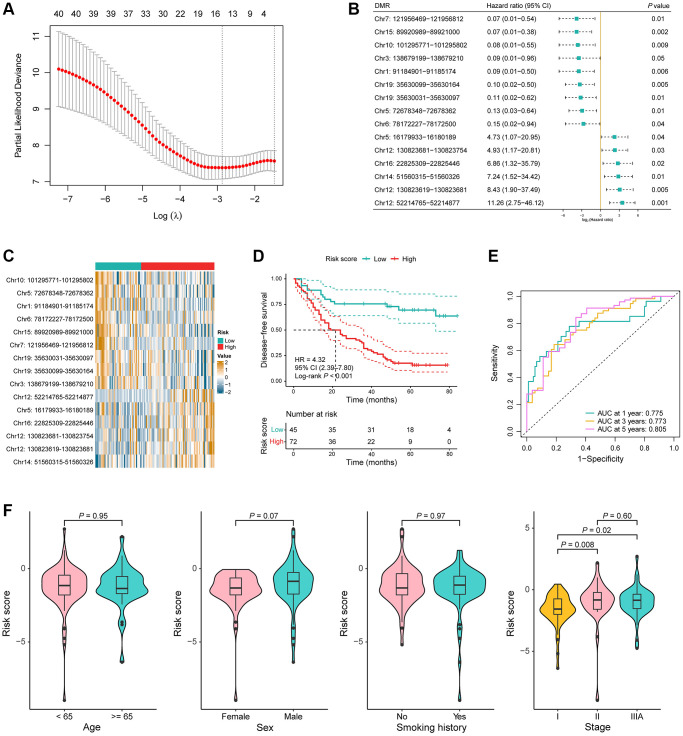
**Construction of a 15-DMR prognostic signature in the model training cohort.** (**A**) LASSO penalized Cox regression analysis yielded 15 DMRs for the recurrence risk predicting model construction. (**B**) Hazard ratio with 95% confidence interval (CI) and *P*-value of the 15 DMRs in the univariable Cox regression analysis for disease-free survival (DFS). (**C**) Heatmap of the methylation value of the 15 DMRs in low-risk (*n* = 45) and high-risk (*n* = 72) groups. (**D**) Kaplan-Meier (KM) curves for DFS in the model training cohort. (**E**) Time-dependent receiver operating characteristic (ROC) curves in the model training cohort. (**F**) Comparison of the risk score in the different clinical subgroups including age, sex, smoking history, and TNM stage.

We then aimed to develop a signature with prognostic value in the model training cohort based on the above identified DMRs. We performed univariable Cox regression analysis and 41 DMRs were found to be related to the DFS of 117 stage I–IIIA LUAD patients, which was followed by LASSO penalized Cox regression analysis. A total of 15 DMRs were ultimately incorporated into the recurrence risk predicting model construction (Figure 3A). Hazard ratio with 95% confidence interval (CI) and *P*-value of the 15 DMRs are shown in Figure 3B. Among them, the methylation value of Chr7: 121956469–121956812 was highly negatively associated with the risk of recurrence (*P* = 0.03, HR = 0.60, 95% CI = 0.38–0.95; Supplementary Figure 2A), while the methylation value of Chr12: 52214765–52214877 was highly positively associated with the risk of recurrence (*P* = 0.02, HR = 1.69, 95% CI = 1.07–2.69; Supplementary Figure 2B). In addition, the gene annotation of the 15 DMRs is summarized in Supplementary Table 2.

LUAD samples in the model training cohort were then segregated into low-risk and high-risk groups utilizing a risk score threshold of −1.44. A comparison was then conducted, examining the methylation values of these 15 DMRs between the two risk groups. The value of Chr10: 101295771–101295802, Chr5: 72678348–72678362, Chr1: 91184901–91185174, Chr6: 78172227–78172500, Chr15: 89920989–89921000, Chr7: 121956469–121956812, Chr19: 35630031–35630097, Chr19: 35630099–35630164, and Chr3: 138679199–138679210—which were negatively associated with the risk of recurrence—were significantly higher in the low-risk group, while the value of Chr12: 52214765–52214877, Chr5: 16179933–16180189, Chr16: 22825309–22825446, Chr12: 130823681–130823754, Chr12: 130823619–130823681, and Chr14: 51560315–51560326—which were positively associated with the risk of recurrence—were significantly higher in the high-risk group (*P* < 0.05; Figure 3C). The KM curve distinctly demonstrated a significantly shorter DFS among high-risk patients compared to their low-risk counterparts (*P* < 0.001, HR = 4.32, 95% CI = 2.39–7.80; Figure 3D). To assess the predictive proficiency for recurrence risk, time-dependent ROC curve analyses were conducted, and the AUC was 0.775 at 1 year, 0.773 at 3 years and 0.805 at 5 years, collectively indicating a robust predictive performance of the DMR signature (Figure 3E). Risk scores had no significant difference between the different age, sex, and smoking history groups, but were obviously higher in stage II and IIIA compared to stage I (stage II vs. stage I: *P* = 0.008, stage IIIA vs. stage I:* P* = 0.02; Figure 3F).

To further assess the prognostic significance of the DMR signature, univariable and multivariable Cox regression analyses were conducted on the most relevant clinical variables of LUAD patients in the training set. Results showed that the risk score was significantly associated with the DFS of patients adjusted for age, sex, smoking history, and TNM stage (univariable: *P* < 0.001, HR = 4.32, 95% CI = 2.39–7.80; multivariable: *P* < 0.001, HR = 4.46, 95% CI = 2.46–8.11; Table 2). In conclusion, the results above suggested that the 15-DMR signature had a good performance in predicting the risk of recurrence for patients with early stage LUAD in the model training cohort.

**Table 2:** Univariable and multivariable Cox regression analysis in the training cohort.

**Parameter**	**Model training cohort (*n* = 117)**			
	**Univariable Cox analysis**		**Multivariable Cox analysis**	
	**HR (95% CI)**	***P-*value**	**HR (95% CI)**	***P*-value**
Age (≥65 vs. <65)	0.95 (0.58–1.58)	0.86	0.92 (0.55–1.55)	0.77
Sex (male vs. female)	1.25 (0.79–1.98)	0.33	1.49 (0.87–2.57)	0.15
Smoking history (yes vs. no)	0.97 (0.62–1.53)	0.90	0.78 (0.46–1.33)	0.36
Stage (stage II–IIIA vs. stage I)	3.77 (2.18–6.54)	<0.001^*^	2.96 (1.68–5.23)	<0.001^*^
Risk score (high vs. low)	4.32 (2.39–7.80)	<0.001^*^	3.37 (1.82–6.23)	<0.001^*^

Note: ^*^represents a statistically significant difference.

### Performance of the 15-DMR signature in the model validation cohort

**Figure 4:**
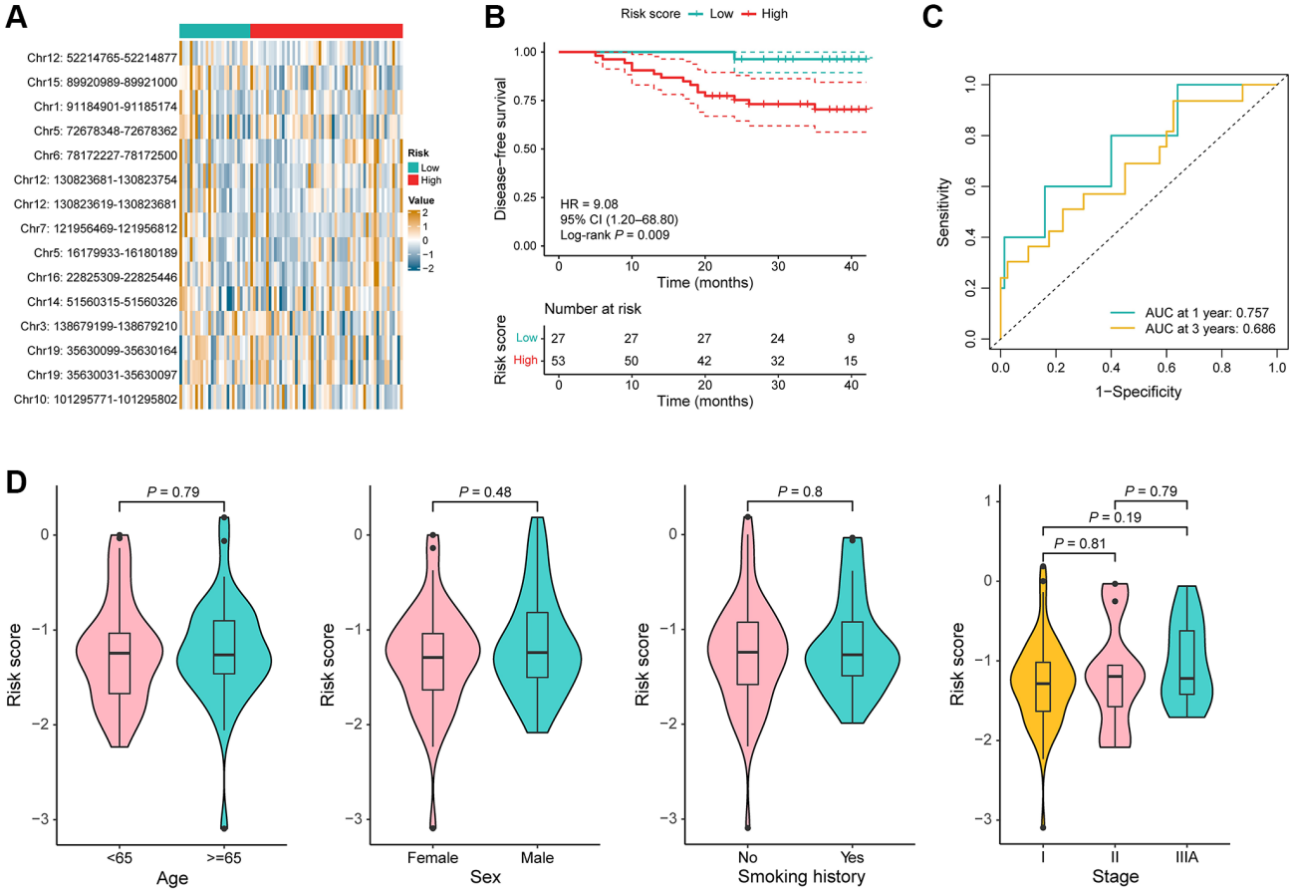
**Performance of the DMR signature in the model validation cohort.** (**A**) Heat map of the methylation value of the 15 DMRs in low-risk (*n* = 27) and high-risk (*n* = 58) groups. (**B**) KM curves for DFS in the model validation cohort. (**C**) Time-dependent ROC curves in the model validation cohort. (**D**) Comparison of the risk score in the different clinical subgroups including age, sex, smoking history, and TNM stage.

To validate the stability of the DMR signature, utilizing the identical algorithm, the risk score for each patient was further computed within the model validation cohort. 80 stage I–IIIA LUAD samples were also stratified into high-risk and low-risk groups by the same cutoff value. Consistent with the training cohort, the value of Chr10: 101295771–101295802, and Chr3: 138679199–138679210 were significantly increased in the low-risk group, while the value of Chr12: 52214765–52214877, Chr12: 130823619–130823681, Chr12: 130823681–130823754, Chr16: 22825309–22825446, and Chr14: 51560315–51560326 were significantly increased in the high-risk group (*P* < 0.05; Figure 4A). In addition, a notable difference was observed that high-risk patients exhibiting significantly shorter DFS compared to their low-risk counterparts (*P* = 0.009, HR = 9.08, 95% CI = 1.20–68.80; Figure 4B). The AUC for predicting risk of recurrence reached 0.757 and 0.686 at 1 and 3 years, respectively (Figure 4C). Risk scores were not statistically different between clinical subgroups (Figure 4D).

Furthermore, both univariable and multivariable Cox regression analysis indicated a robust association between the risk score and the likelihood of recurrence in patients with early stage LUAD (univariable: *P* = 0.03, HR = 9.08, 95% CI = 1.20–68.80; multivariable: *P* = 0.04, HR = 8.50, 95% CI = 1.08–66.56; Table 3). In addition to risk score, as expected, TNM stage is an independent risk factor for prognosis. Thus, a nomogram incorporating the risk score and stage was devised to better identify patients of their risk of recurrence (Supplementary Figure 3). Altogether, we developed and validated a 15-DMR signature that was associated with prognosis of patients with LUAD.

**Table 3:** Univariable and multivariable Cox regression analysis in the validation cohort.

**Parameter**	**Model validation cohort (*n* = 80)**			
	**Univariable Cox analysis**		**Multivariable Cox analysis**	
	**HR (95% CI)**	***P*-value**	**HR (95% CI)**	***P*-value**
Age (≥65 vs. <65)	2.02 (0.73–5.56)	0.17	1.82 (0.63–5.27)	0.27
Sex (male vs. female)	2.46 (0.86–7.09)	0.10	2.56 (0.59–11.07)	0.21
Smoking history (yes vs. no)	2.25 (0.84–6.00)	0.11	0.88 (0.22–3.57)	0.86
Stage (stage II–IIIA vs. stage I)	5.18 (1.91–14.04)	0.001^*^	4.73 (1.63–13.69)	0.004^*^
Risk score (high vs. low)	9.08 (1.20–68.80)	0.03^*^	8.50 (1.08–66.56)	0.04^*^

Note: ^*^represents a statistically significant difference.

## DISCUSSION

Extensive research on genetic mutations has shed crucial mechanistic insights into the tumorigenic processes of diverse tumors. However, it is increasingly evident that epigenetic alterations also occupy a pivotal role in cancer progression. Among these, alterations in DNA methylation patterns stand out as the most quantifiable and well-characterized epigenetic changes in cancer [30]. In addition, the reversibility of DNA methylation presents a potential to facilitate the monitoring of therapeutic outcomes [31], suggesting it could pioneer a novel approach for predicting LUAD recurrence and enhancing its prognostic outcomes.

In the process of carcinogenesis, certain genes can be activated due to the loss of CpG island methylation, often coupled with the absence of maternal or paternal imprinting [32]. However, a more prevalent alteration observed in CpG islands is hypermethylation, serving as a pivotal mechanism for gene inactivation, typically involving those genes that typically play a crucial role in negatively regulating cell growth. The well-established connection between cytosine residue methylation in promoters and the subsequent transcriptional silencing of genes has been extensively documented [33–35]. Virtually all types of human cancers investigated exhibit hypermethylation of CpG islands [36, 37], including lung cancer [38, 39].

In our study, 468 DMRs were discovered between the early stage LUAD samples and adjacent lung samples in the marker selection cohort. Most of the DMRs were located in the CpG islands and transcription start site of promoter with hypermethylation, which was consistent with previous studies [38, 39]. Of all the DMRs, the methylation levels of 41 DMRs were significantly associated with the DFS of patients with LUAD. A total of 15 DMRs were subsequently utilized to construct a signature for predicting the recurrence risk of LUAD patients in the training cohort, and the risk score generated from the DMR signature was negatively associated with the DFS of patients. The prognostic effect was subsequently verified in a validation cohort.

The 15 DMRs included in the signature corresponded to the following genes—*PIWIL1, HTR1B, HS3ST2, NKX2-3, FIGNL2, FXYD1, FEZF1, TRIM9*, etc., among which several genes have been investigated for the role in LUAD. *PIWIL1* is a member of *PIWI* family and can bind to *PIWIL*-interacting RNAs during spermatogenesis [40]. Increasing evidence indicates that *PIWIL1* is frequently expressed in diverse cancer types, including lung cancer, hinting at its potential oncogenic roles in cancer development or progression [41–44]. A study uncovered an association between elevated *PIWIL1* expression and reduced survival rates among patients with LUAD. Overexpression of *PIWIL1* promotes proliferation, invasion, and migration in LUAD cells, and conversely, its downregulation exhibits opposite effects [45]. In our study, two DMRs located in the 1st intron of *PIWIL1* exhibited hypermethylation in the high-risk group, suggesting that the intron methylation levels of *PIWIL1* might be involved in regulating *PIWIL1* expression. A prognostic signature of 8 genes including *MYBPH, GUCA2A, AZGP1, INPP5J, SPIB, SLC15A1, HTR1B,* and *TNFSF11*, was developed to determine the risk of recurrence in LUAD. This model revealed that patients with higher risk scores exhibited significantly shorter recurrence-free survival durations compared to those with lower risk scores [46]. In this study, the expression of *HTR1B* was observed to be positively correlated with the recurrence, which is consistent with our result that *HTR1B* was hypomethylated in high-risk patients. *HS3ST2*, a constituent of the heparan sulfate biosynthetic enzyme family, demonstrates heparan sulfate glucosaminyl 3-O-sulfotransferase activity, essential for modifying glycosaminoglycan chains. Notably, hypermethylation of *HS3ST2* has been documented in diverse cancer types, including breast cancer [47, 48], colorectal cancer [47, 49], gastric cancer [50] and lung cancer [47], etc. It was reported that patients with *HS3ST2* hypermethylation had poor overall survival (OS) in 193 stage I-II NSCLCs, upon adjustment for factors such as sex, age, tumor size, differentiation, adjuvant therapy, and recurrence [51]. Similar result was observed in our study that hypermethylated *HS3ST2* was associated with shorter DFS of patients with LUAD.

DNA methylation in limited CpG sites has been studied in predicting the prognosis of LUAD patients. Three methylation CpG sites (cg15386964, cg14517217, and cg18878992) that were associated with the prognosis of LUAD were selected in TCGA-LUAD set, which was followed by a validation in Chinese population. The risk of mortality in LUAD patients escalated with a progressive rise in the methylation signature, derived from the assessment of three methylation site levels [52]. Another study reported a prognosis signature including six DNA methylation sites based on the TCGA database. The discrimination effect of this DNA methylation signature for the OS of LUAD patients was obvious [53]. A prognostic signature encompassing 16 CpG sites was devised and validated utilizing DNA methylation, RNA-seq, and clinical data sourced from LUAD patients in the TCGA database. This study showed stable prognostic performance to assess the OS of patients with LUAD in the stratified cohorts [54]. In sum, most of the DNA methylation prognostic signatures of limited CpG sites were developed from the methylation microarray data of TCGA, and validated in the methylation data generated from another platform. However, in our study, the DNA methylation signature was constructed based on DMRs, which have been unveiled to hold greater significance in cancer detection than individual CpG sites [25], and might be more appropriate markers in predicting prognosis. The marker selection, model training, and performance validation of the DMR signature were totally carried out in three in-house clinical cohorts and a uniformed platform and sequencing panel was used, further improving the reliability of our results. Further, we focused on the recurrence risk of LUAD patients after surgery, thus DFS was included in our study. From what has been discussed above, our study provides new insights on enhancing the clinical management of early-stage LUAD by refining risk stratification utilizing methylation profile.

However, this study has several limitations that need to be clearly addressed. First, besides surgery, other detailed information on adjuvant treatment for the three cohorts was not available in this study. Second, the sample size of the marker discovery cohort is limited. The DMR signature is yet to be optimized to be more feasible for clinical application. Third, further validation, such as prospective studies with larger sample sizes, is still required to validate the DMR signature and assess its reliability in different settings. Moreover, efforts should be directed toward integrating the DMR signature with complementary prognostic markers, encompassing clinicopathological parameters, genomic mutations, and gene expression profiles, to optimize the predictive ability of the risk model.

## CONCLUSION

To sum up, the DMRs between LUAD and normal lung samples were screened out, and a prognostic signature for recurrence risk prediction was determined based on 15 DMRs in early stage LUAD patients. The DMR signature shows commendable performance in predicting the risk of recurrence in LUAD patients both in model training and validation cohorts, which might hold significant potential for understanding the molecular underpinnings of LUAD relapse, providing risk stratification of patients, and establishing future monitoring plans.

## Supplementary Materials

Supplementary Figures

Supplementary Table 1

Supplementary Table 2
