# Supplementary material for: A differentially-methylated-region signature predicts the recurrence risk for patients with early stage lung adenocarcinoma
Source: Aging (Albany NY). 2024 Nov 18;16(21):13323–39. doi: 10.18632/aging.206139 (PMC11719112; doi:10.18632/aging.206139)
Supplement: Supplementary Figures [file aging-16-206139-s001.pdf]

SUPPLEMENTARY FIGURES

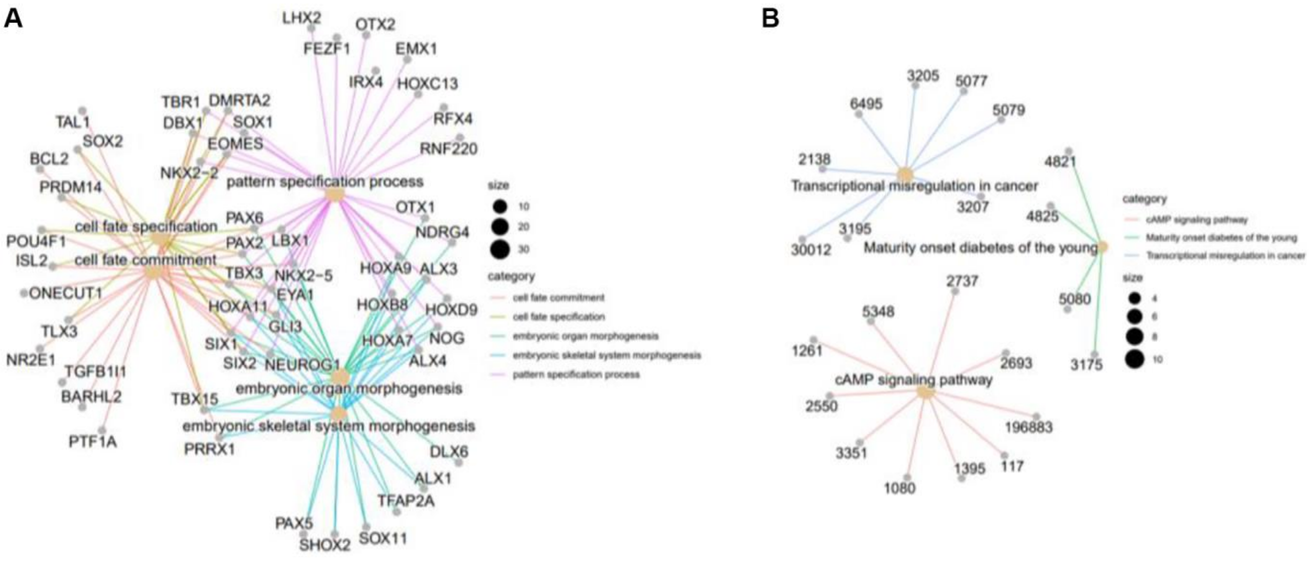

Supplementary Figure 1. GO analysis (**A**) and KEGG analysis (**B**) of the genes corresponding to the DMRs.

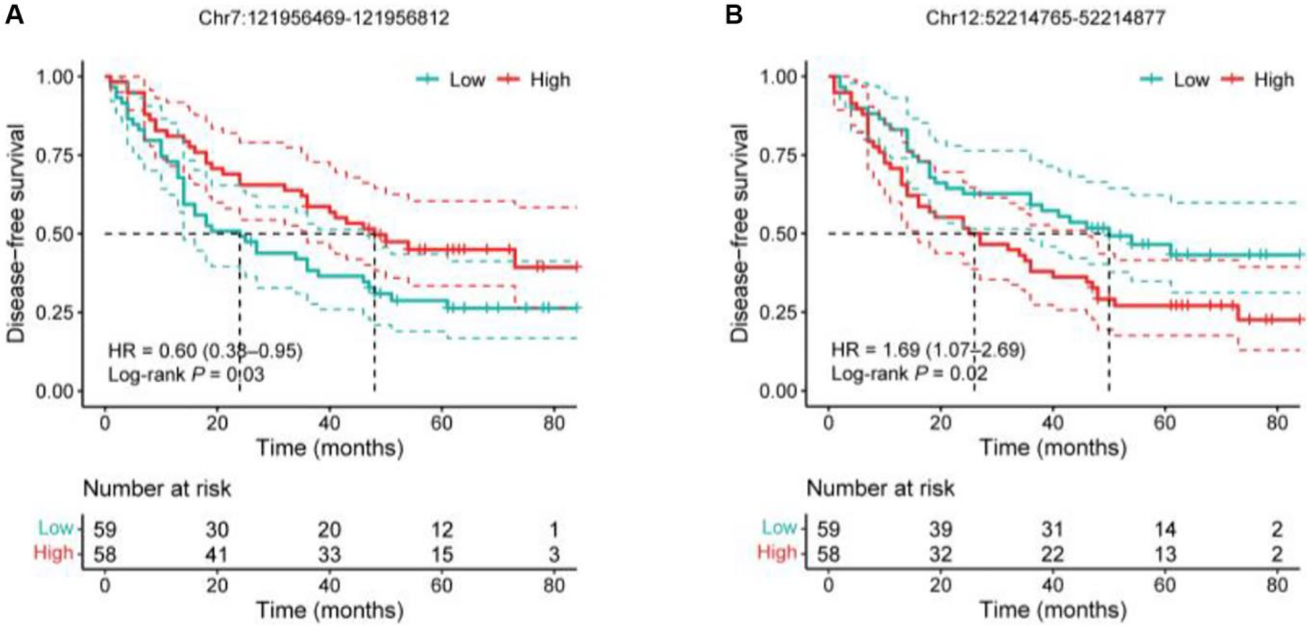

Supplementary Figure 2. Survival analysis of the DMR with the minimum HR (**A**) and maximum HR (**B**) for DFS.

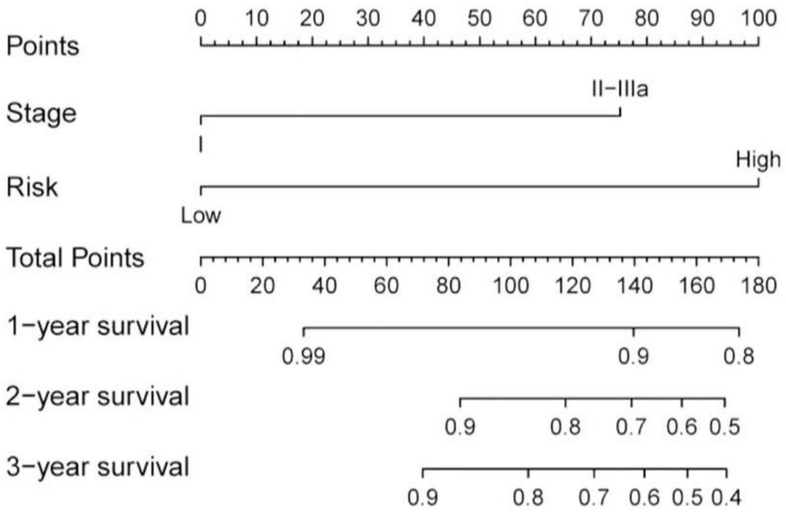

Supplementary Figure 3. Nomogram integrating risk score and TNM stage for DFS prediction in the validation cohort.
