# Supplementary material for: A differentially-methylated-region signature predicts the recurrence risk for patients with early stage lung adenocarcinoma
Source: Aging (Albany NY). 2024 Nov 18;16(21):13323–39. doi: 10.18632/aging.206139 (PMC11719112; doi:10.18632/aging.206139)
Supplement: Supplementary Table 2 [file aging-16-206139-s003.pdf]

SUPPLEMENTARY TABLE

Supplementary Table 2. Gene annotation of the 15 DMRs included in the prognostic signature.

DMR	Gene	Location
Chr1:91184904-91185172	BARHL2	Intergenic
Chr10:101295774-101295800	NKX2-3	3'UTR (NM_145285, exon 2 of 2)
Chr12:130823622-130823679	PIWIL1	intron (NM_001190971, intron 1 of 19)
Chr12:130823684-130823752	PIWIL1	intron (NM_001190971, intron 1 of 19)
Chr12:52214768-52214875	FIGNL2	exon (NM_001013690, exon 2 of 2)
Chr14:51560318-51560324	TRIM9	intron (NM_052978, intron 1 of 6)
Chr15:89920992-89920998	MIR9-3HG	promoter-TSS (NR_015411)
Chr16:22825312-22825444	HS3ST2	promoter-TSS (NM_006043)
Chr19:35630034-35630095	FXYD1	promoter-TSS (NM_001278718)
Chr19:35630102-35630162	FXYD1	promoter-TSS (NM_001278718)
Chr3:138679202-138679208	FOXL2NB	Intergenic
Chr5:16179936-16180187	44631	promoter-TSS (NR_149044)
Chr5:72678351-72678360	LINC02230	Intergenic
Chr6:78172230-78172498	HTR1B	exon (NM_000863, exon 1 of 1)
Chr7:121956472-121956810	FEZF1	Intergenic
